# Carbohydrate based meningococcal vaccines: past and present overview

**DOI:** 10.1007/s10719-021-09990-y

**Published:** 2021-04-27

**Authors:** Francesco Berti, Maria Rosaria Romano, Francesca Micoli, Roberto Adamo

**Affiliations:** 1 GSK, Siena, Italy; 2 GSK Vaccines Institute for Global Health, Siena, Italy

## Abstract

*Neisseria meningitidis* is a major cause of bacterial meningitidis worldwide. Children less than five years and adolescents are particularly affected. Nearly all invasive strains are surrounded by a polysaccharide capsule, based on which, 12 *N. meningitidis* serogroups are differentiated. Six of them, A, B, C, W, X, and Y, cause the vast majority of infections in humans. Mono- and multi-valent carbohydrate-based vaccines against meningococcal infections have been licensed or are currently in clinical development. In this mini-review, an overview of the past and present approaches for producing meningococcal glycoconjugate vaccines is provided.

## Introduction

“Meningitis” is an inflammation of the membranes (meninges) and/or cerebrospinal fluid (CSF) surrounding and protecting brain and spinal cord. Along with *Streptococcus pneumoniae* and *Haemophilus influenzae* type b (Hib), *Neisseria meningitidis* is a major cause of bacterial meningitis. *N. meningitidis* is a respiratory pathogen, cause of infection that can spread through respiratory secretions. After pharyngeal colonization, the pathogen can cross the mucosa and enter the blood reaching the meninges, causing meningitis. Typical symptoms of bacterial meningitis include headache, stiff neck, fever, chills, malaise, and prostration [1].

Until the beginning of the second millennium, over 1.2 million cases of bacterial meningitis were estimated to occur worldwide each year [2]. The incidence and case-fatality rates for bacterial meningitis vary by region, country, pathogen, and age group. Without treatment, the case-fatality rate can be as high as 70 %, and one in five survivors of bacterial meningitis may be left with permanent sequelae including hearing loss, neurologic disability, or loss of a limb. Meningococcal infections can be complicated by purpuric rash, purpura fulminans, arthritis, myocarditis, pericarditis, endophthalmitis, or pneumonia. *N. meningitidis* can also cause a severe bacteremia, called meningococcemia [3].

*N. meningitidis*, which was identified as one of the causative agents of bacterial meningitis by Weichselbaum in 1887, is a gram-negative diplococcus bacterium which can either be encapsulated or unencapsulated [1]. Nearly all invasive strains are surrounded by a polysaccharide capsule. Based on the capsular polysaccharide structure, 12 *N. meningitidis* serogroups are differentiated. Six of them cause the great majority of infections in humans: A, B, C, W, X, and Y (https://www.cdc.gov/vaccines/pubs/surv-manual/chpt08-mening.html). Incidence rates of *N. meningitidis* are generally highest in children less than five years of age and in adolescents. Neonates are relatively protected against meningococcal disease as a result of passive acquisition of transplacental maternal antibodies [4].

The worldwide distribution of serogroups of *N. meningitidis* is variable. In the Americas, Europe, and Australia, serogroups B and C are the most common, while serogroup A causes the majority of disease in Africa and Asia [5, 6]. Recently emergence of other serogroups, such as C in China [7] or serogroup Y in North America has been reported [8, 9]. The region where the incidence was higher, until the recent introduction of vaccination, is the sub Saharan Africa, known as the “meningitis belt” [6], where incidence rates can be greater than 1,000 cases per 100,000 population. Across the meningitis belt, at least 350 million people are at risk for meningitis during annual epidemics. Meningitis epidemics are generally caused by serogroup A, although outbreaks have also been caused by serogroups C, W, and X [10–12], even in recent times. This minireview aims at providing an historical overview of the development of commercialized carbohydrate based meningococcal vaccines and present the more recent approaches explored at preclinical level.

## Historical overview

**Fig. 1:**
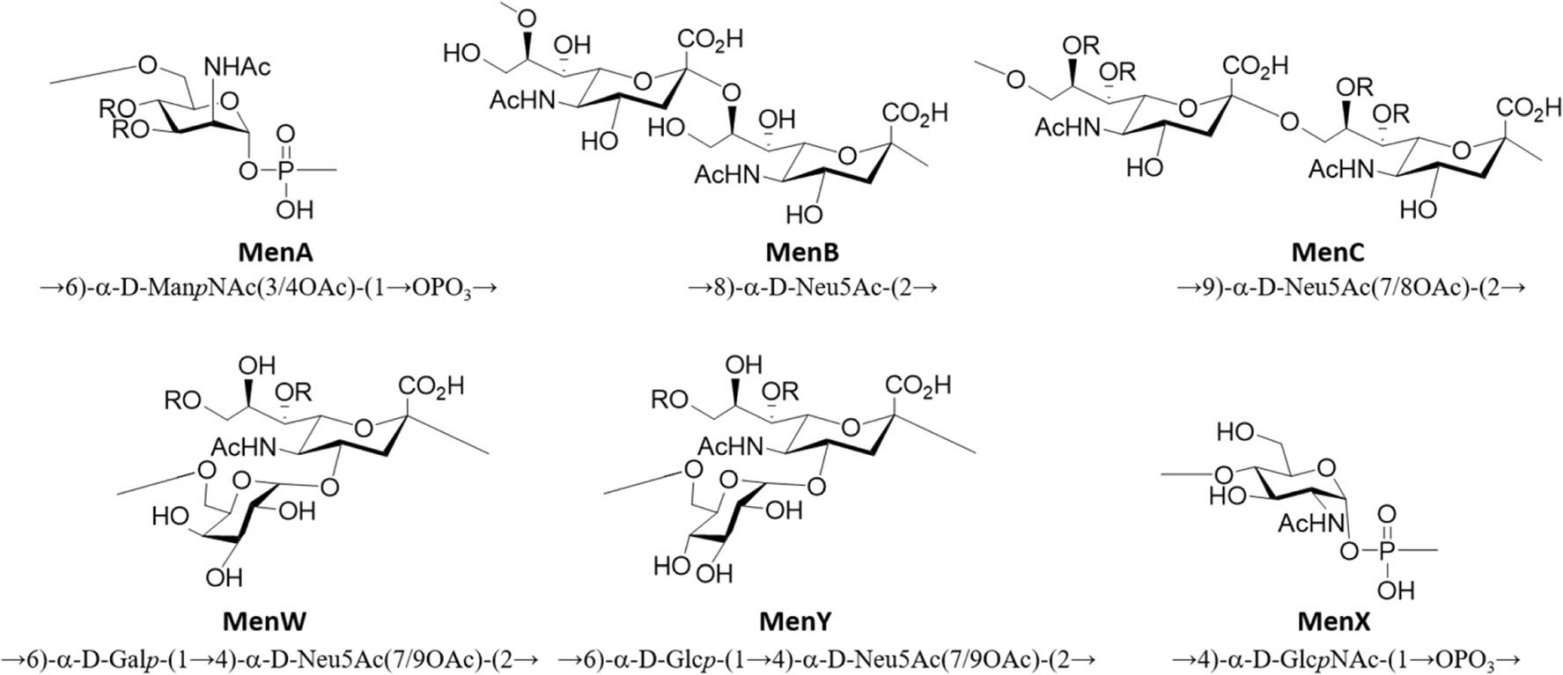
Chemical structure of meningococcal capsular polysaccharide repeating units from serogroups relevant for the disease. Some structures are partially O-acetylated (R = Ac)

The chemical structure of the six prevalent meningococcal serogroups (Men) is described in Fig. 1  [13, 14].

Meningococcal polysaccharide vaccines became available since the 1970s [15]. Tetravalent serogroup A, C, W and Y polysaccharide vaccines (Mencevax, GSK Vaccines; Menomune, Sanofi Pasteur) were licensed in the 1980s [16].

However, limitations of these vaccines, including poor immunogenicity in children below the age of two, lack of immunological memory and, in certain cases, hyporesponsiveness, led to the development of meningococcal conjugate vaccines, where the capsular polysaccharide is covalently linked to a carrier protein [17, 18].

The first meningococcal conjugate vaccines were introduced in 1999 in the United Kingdom, to prevent the serogroup C related infection [19]. Three vaccines (Meningitec, Nuron Biotech, formerly Pfizer; Menjugate, GSK Vaccines, formerly Novartis Vaccines; NeisVac-C, Pfizer, formerly Baxter) were developed, licensed and introduced as part of routine immunization of all children aged > 4 months to < 18 years [19]. The vaccine effectiveness was of 81 % overall and 92–97 % in teenagers, leading to a reduction of the number of deaths from 67 to 1999 to 5 in 2001 [20]. Importantly, the experience of MenC vaccination in UK adolescents, which is the age when most of transmission occurs, underscored the effectiveness of vaccination on reduction of carriage and transmission of the infection to unvaccinated individuals, the so called herd immunity [21]. Similar evidence was later confirmed also in other countries where different immunization schedules were applied.

Bivalent Men C/Hib (Menitorix, GSK Vaccines) and a trivalent Men CY/Hib (MenHibrix, GSK Vaccines) conjugate vaccines have been also licensed [22]. MenHibrix vaccine has been recently discontinued at GSK Vaccines. Menitorix was specifically developed for national immunization programs in UK and Australia.

The currently landscape of meningococcal conjugate vaccine is summarized in Table 1. In the last decades, three tetravalent serogroups ACWY conjugates were also developed and launched in several countries: Menactra (MenACWY-DT; Sanofi Pasteur) was launched in 2005, followed by Menveo (MenACWY-CRM_197_; GSK Vaccines, formerly Novartis Vaccines) in 2010 and Nimenrix (MenACWY-TT; Pfizer, formerly GSK Vaccines) in 2012 [23]. MenACWY-DT and MenACWY- CRM_197_ are licensed in the United States for those aged 9 months through 55 years and 2 months through 55 years, respectively [24]. MenACWY-CRM_197_ is also licensed in Europe for those aged ≥ 2 years [24]. MenACWY-TT was first licensed in Europe in 2012 for children aged > 12 months and in 2016 for infants aged ≥ 6 weeks [23]. Recently, MenQuadfi vaccine (MenACWY-TT; Sanofi Pasteur) has been also licensed in US for > 12 months subjects and its launch in EU is expected in 2021 (https://www.ema.europa.eu/en/medicines/human/EPAR/menquadfi). This vaccine replaced Menactra which contained polysaccharides conjugated to Diphtheria Toxoid, which was FDA approved for prevention of invasive meningococcal disease in individuals 9 months through 55 years of age (https://www.fda.gov/vaccines-blood-biologics/vaccines/menactra).

**Table 1:** Multivalent meningococcal conjugate vaccines licensed or in clinical trials worldwide. The multivalent conjugate vaccines containing at least two Men serogroups are listed here

Commercial trade name/clinical trial phase	Target serogroup	Manufacturer
*MengNa Kang*	AC (a)	Beijing Luzhu (subsidiary of ChongquingZhifei)
Nao Man King		Xiangrui (also known as Beijing Sanroad)
Wo Er Kang		Yunnan Walvax
MeningACon		BeijingZhifei Lvzhu Biopharmaceutical
N/A		Royal Wuxi
Menactra	ACWY	Sanofi Pasteur
Menveo		GSK Vaccines
Nimenrix		Pfizer
MenQuadfi		Sanofi Pasteur
Phase 1/2	ACWYX	Serum Institute of India/PATH
Phase 3	ABCWY (b)	GSK Vaccines
Phase 3		Pfizer

(a) For national vaccination; (b) composed of glycoconjugates combined with other antigens

To finally eliminate epidemic meningitis in the meningitidis belt, a monovalent conjugate vaccine (MenAfriVac, Serum Institute of India Ltd) was successfully developed, tested, licensed [25] and distributed as part of the Meningitis Vaccine Project (MVP), a Gates Foundation funded partnership between PATH (Program for Appropriate Technology in Health) and the World Health Organization (WHO). After the implementation of vaccination, a dramatic reduction of the infection until almost disappearance has been observed [26].

Of note, immunization with the meningococcal serogroup B polysaccharide antigen, which is composed of the →8)-α-D-Neu5Ac-(2→ repeating unit (Fig. 1) has proven unfeasible due to the structural similarity with protein-attached glycans expressed in the fetus associated with neural development, which results in immune tolerance [27]. Attempts to overcome the immune tolerance to this polysaccharide have been made [28], and the use of an N-propionyl sialyl conjugate vaccine led to antibodies which were either not cross-reactive or minimally cross-reactive with purified polysaccharide or human polysialic acid antigens [29]. Although other strategies have been shown premise to induce cross reactive antibodies with MenB capsule in mice [30], safety concerns have remained serious. For this reason, this specific serogroup has been targeted through different approaches, such as the use of Outer Membrane Vesicles (OMVs) containing outer membrane proteins (OMPs), chemically treated by detergent to reduce reactogenicity, or recombinant proteins selected by reverse vaccinology [31–33].

Vaccines containing recombinant proteins only (Trumenba, Pfizer), or recombinant proteins combined with OMVs (Bexsero, GSK Vaccines), or containing both meningococcal polysaccharide of serogroups C and OMPs of serogroup B (VA-Mengoc-BC, Finley Institute) have been developed and licensed. To further expand this topic we suggest some reviews [15, 32, 34].

Current research is aimed at further broadening the vaccine coverage by pentavalent formulations. Particularly, the monovalent MenA MenAfriVac vaccine is being extended to a pentavalent ACWYX vaccine [35], whereas licensed ACWY conjugates have been proposed as pentavalent combinations with serogroup B antigens (Bexsero and Trumenba) (https://clinicaltrials.gov/ct2/show/NCT02285777?term=ABCWY&draw=2&rank=1, https://clinicaltrials.gov/ct2/show/NCT04440163?term=ABCWY&draw=2&rank=8).

A Phase 1 clinical trial conducted in 2016-17 on sixty adults randomized to receive a single dose of the pentavalent MenACWYX (Alum-adjuvanted), as compared to MenACWYX (non-adjuvanted) or MenACWY Menactra, confirmed a high safety profile of the pentavalent vaccine, with no serious adverse effects recorded [35].

The non-adjuvanted vaccine elicited high titers of bactericidal antibodies, which were not inferior to the adjuvanted formulation. In addition to the strong MenX immunogenicity induced, the response against the other serogroups was not inferior in comparison to the control vaccine Menactra. A Phase 2 study in Malian 12–24 month old children started in late 2017 of which results are not yet available.

To simplify the immunization schedule, a single combination meningococcal vaccine that includes the antigens of the licensed MenACWY and MenB vaccines would be recommended. For this reason, the Trumemba vaccine, which contains two recombinant lipidated MenB factor H binding protein (fHbp) variants, has been combined into the corresponding pentavalent MenACWYB vaccine formulation, which is currently in Phase 3 (NCT04440163) (https://clinicaltrials.gov/ct2/show/NCT04440163?term=ABCWY&draw=2&rank=8). Likewise, a combination of MenACWY-CRM_197_ conjugate antigens, contained in Menveo vaccine, and the tetravalent Alum-adjuvanted Bexsero vaccine (containing fHbp; *Neisseria* adhesin A, NadA; Neisserial Heparin Binding Antigen, NHBA, OMVs, from the New Zealand outbreak strain NZ98/254) is currently under clinical investigation. In a Phase 2 randomized study, designed to study effect of two or three vaccine doses, adolescents aged 10–18 years received either 3 MenABCWY doses (MenABCWY group), on a 0, 2, 6-month schedule, while the control group received a single MenACWY-CRM_197_ dose at month 2 and placebo at 0, 6-month. The vaccine gave robust immune responses to antigen-specific test strains for each serogroup already at the second dose, while no reactogenicity or safety concern arose during the study [36]. The Phase 3 (NCT04502693) for this candidate is also in progress (https://clinicaltrials.gov/ct2/show/NCT04502693?term=abcwy+gsk&draw=2&rank=1).

## Conjugation chemistry of meningococcal vaccines

Meningococcal carbohydrate-based vaccines commercialized or under development are made by conjugation of extracted polysaccharides to protein. Three carrier proteins (TT, Tetanus Toxoid; DT, Diphtheria Toxoid; CRM_197_, Cross Reacting Material 197) have been used for this type of vaccines [37]. The toxoids DT and TT are detoxified by chemical treatment with formaldehyde, while CRM_197_, a nontoxic mutant of diphtheria toxin, is expressed as genetically detoxified protein from *Corynebacterium diphtheriae* C7(b197) [38], and, more recently, as recombinant protein in *E. coli * [39].

**Fig. 2:**
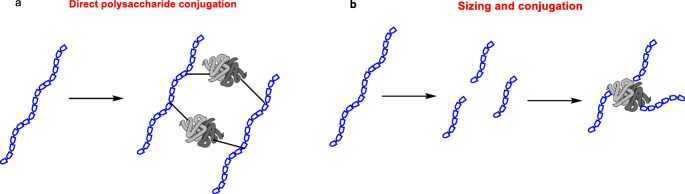
Different modes for the preparation of conjugates from polysaccharides: (**a**) direct conjugation of naturally extracted carbohydrates and (**b**) sizing of the polysaccharide prior to conjugation

The polysaccharide can be used as it is to be randomly linked to the protein or sized prior to conjugation through the terminal end, giving rise to two types of structurally different conjugates, as depicted in Fig. 2.

**Fig. 3:**
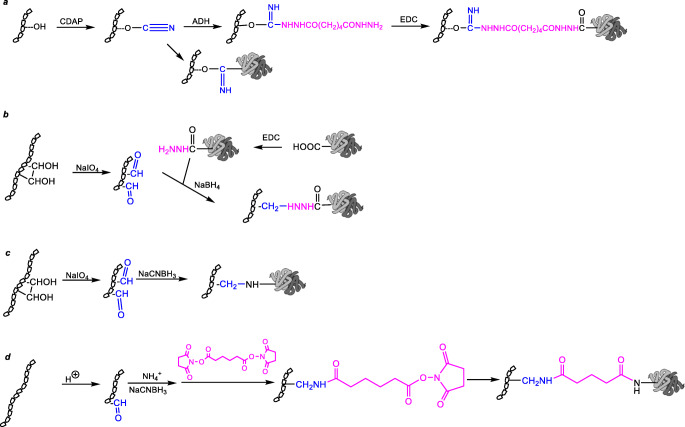
Most common conjugation strategies exploited for meningococcal glycoconjugates

An example of the first strategy is the MenACWY-TT vaccine [23, 40], developed by GSK and now part of Pfizer pipeline. The hydroxyl groups of MenW and Y polysaccharides are preactivated with CDAP reagent (1-cyano-4-dimethylaminopyridinium tetrafluoroborate) and directly coupled to TT carrier protein. CDAP, cyanylation reagent, can be replaced by cyanogen bromide (CNBr) that does not require as low pH as CDAP. Differently, the hydroxyl groups of MenA and C polysaccharides are activated with CDAP to be coupled with a spacer molecule (adipic acid dihydrazide, ADH) and finally conjugated to TT by EDC chemistry (Fig. 3a) [41].

Also the MenA conjugate component of the MenAfriVac vaccine is obtained via polysaccharide conjugation: the carboxyl groups of aspartic and glutamic acid residues of TT carrier protein are activated by treatment with hydrazine (NH_2_-NH_2_) in the presence of the coupling reagent EDC to form carboxylhydrazide groups (Fig. 3b) [42]. The MenA polysaccharide is oxidized at the position C-3 and C-4 of the non O-acetylated repeating units. Incubation of the activated protein and polysaccharide enables the formation of a Schiff’s base, which is finally reduced to a stable covalent linkage by treatment with sodium borohydride (NaBH_4_) (Fig. 3b).

An example of polysaccharide sizing is the method applied by Baxter to break down the polymer by sodium periodate oxidation of vicinal diols with concomitant generation of aldehydes for conjugation to the carrier protein via reductive amination (Fig. 3c). This methodology has been used for the preparation of the licensed Meningitec vaccine [17], where the periodate oxidation of MenC occurs at the level of the non O-acetylated hydroxyl group of the glycerol chain of the sialic acid repeating units.

For the preparation of the Menveo ACWY vaccine, the polysaccharide is subjected to controlled acid hydrolysis, generating a polydispersion of oligosaccharides, which is then chromatographically sized to obtain a more defined length, and activated *via* reductive amination for coupling to a bis-N-hydroxysuccinimidyl adipate linker (Fig. 3d). The formed active half-ester is finally conjugated to CRM_197 _ [43–46]. Alternatively, a highly immunogenic MenX-CRM_197_ conjugate at preclinical level has been generated by reductive amination of the end terminal aldehyde in the presence of a di-hydrazine linker for following coupling to the protein with the bis-N-hydroxysuccinimidyl adipate spacer [47].

Other methodologies for depolymerization can be applied, including hydrogen peroxidation followed by an activation step to provide chemical groups capable of reacting with the carrier protein [48].

## New approaches under preclinical investigation

Innovative designs for meningococcal vaccines aiming at improvement of current formulations based on classic polysaccharide conjugates or exploitation of new platforms for pathogen free production of well-defined carbohydrate antigens have been proposed.

Conjugation of oligosaccharides from two diverse serogroups, namely MenA and C, to the same carrier protein has been shown feasible. Although in mice the immune response was lower for MenA at the second injection, the bivalent conjugate induced high titers of bactericidal antibodies against the two different serogroups after the third administration [49].

It needs to be mentioned that in the animal model MenA seems to be the weakest among the different polysaccharides in terms of immunogenicity, and the one suffering from carrier epitope suppression to preexisting immunity to the carrier, particularly CRM_197_ and TT [50]. The use of a carrier for MenA different than the other serogroups has been shown to overcome this issue [51]. Some proteins like the pneumococcal Spr91-2021 have proven to be particularly efficient as carriers for MenA in mice [51].

Meningococcal polysaccharides have been also conjugated to nanoparticle carriers to improve the onset or persistence of antibodies. Conjugation of MenC polysaccharide to full-length hepatitis B core antigen (Hbc) virus-like particles through heterobifunctional polyethylene glycol linkers led to the generation of anti-carbohydrate IgG about 10 times higher than the unconjugated polysaccharide with a significant boosting effect [52]. HBc conjugation induced also shift to a Th1 cellular immune type response, as assessed by the increased IgG2a subclass production.

Genetically detoxified OMVs (GMMA) from MenB have also been shown to act as potent carrier for MenA and MenC polysaccharides, giving levels of murine bactericidal antibodies superior to CRM_197_ already at the first dose [53].

In the last years, chemoenzymatic synthesis has been successfully applied to cell-free production of different meningococcal serogroups polysaccharide thanks to the identification of a variety of enzymes involved in bacterial capsular biosynthesis demonstrating the feasibility of enzymatic elongation of polysaccharide *in vitro* [54–59].

In 2014, a soluble version of the polymerase was developed for the *in vitro* synthesis of the polysaccharide *N. meningitidis serogroup* A. The molecular cloning and functional expression of the three enzymes (UDP-GlcNAc-2-epimerase, CsaA; poly-ManNAc-1-phosphate-transferase, CsaB; O-acetyltransferase, CsaC) were identified as part of the capsular biosynthesis complex in MenA and represent the minimal number of enzymes needed to produce *in vitro* structures identical to the native polysaccharide and immunologically active [54]. Recent studies on enzymatic O-acetylation mechanism in the ManNAc unit have shown that O-acetylation occurs in the O3 position due to enzymatic transfer with rigid regioselectivity of the O-acetyltransferase CsaC enzyme, while acetylation in the O4 position occurs only by migration [60]. This work set the foundations for the fully enzymatic production of this polysaccharide.

For the production of MenX oligomers, initially polymerase CsxA was exploited to elongate naturally derived acceptors and then generate glycoconjugates [56]. Subsequently, a highly efficient chemoenzymatic approach was designed, combining the chemical synthesis of a trisaccharide acceptor, equipped with a linker for conjugation, with fast elongation mediated by a column immobilized truncated form of the polymerase CsxA [58]. Using GlcNAc-UDP donor, a structure with an average length of 12 repeating units was assembled one-pot and purified to be then conjugated to CRM_197_. Vaccination of mice elicited high level of bactericidal antibodies comparably to a previous conjugate made from extracted material. Compared to conventional synthetic protocols, this methodology proved very expeditious and drastically reduced the number of purification steps to achieve the oligomers.

A chemoenzymatic method was also developed for the biosynthesis of the polysaccharide *N. meningitidis serogroup C*, using chemically synthesized lactosides as substrates for the recombinant sialyltransferase enzymes from *N. meningitidis serogroup C*. The resulting oligosialic acids had the same structure as the *Neisseria* polysaccharide capsule except for the addition of a useful end terminal azide aglycone for conjugation to a carrier protein. The glycoconjugates, tested in *in vivo* model, elicited a specific anti meningococcal immune response [59].

Recently, studies on MenW capsular polymerase (NmSiaDW) allowed identification of 4-azido-4-deoxy-N-acetylmannosamine (ManNAc4N3) and 6-azido-6-deoxy-N-acetylmannosamine (ManNAc6N3) as suitable substrates for the synthesis of N-acetyl MenW oligosaccharides containing 7-O-acetyl-N-acetylneuraminic acid and/or 9-O-acetyl-N-acetylneuraminic acid [61]. A strategy based one-pot use of multiple enzymes was established to assemble NAc-oligosaccharides up to a length of pentasaccharide starting from sugar nucleotide donors generated *in situ* from readily available monosaccharides containing azide groups to mask the N-acetyl moieties. Overall, these studies suggest that chemoenzymatic approaches can be very versatile and could be expanded to other serogroups.

Synthetic routes have been exploited as alternative sources to provide the oligosaccharides for conjugation. This approach, which would avoid pathogen fermentation providing well defined carbohydrates with high purity, has been applied to MenA [62–64], C [65, 66], W [67] and X [68, 69] polysaccharides, of which small size oligosaccharides have been described.

Synthetic tetramer and octamer of MenC polysaccharide have been synthesized and conjugated with TT to induce in a mouse model anti-MenC polysaccharide IgG as well as serum bactericidal titers at levels comparable to those elicited by the licensed vaccine [70]. The increase in length of synthetic oligomers from tetramer to octamer did not appear to be necessary to increase immunogenicity.

**Fig. 4:**
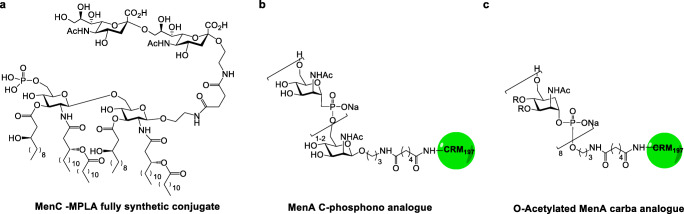
Novel meningococcal synthetic conjugates

A fully synthetic MenC sugar antigen linked to a lipid A carrier MPLA has also been proposed, showing to elicit good levels of bactericidal antibodies (Fig. 4a) [71].

MenA polysaccharide has been attractive biomolecule for the development of biologically active glycomimetics. In particular, the development and manufacture of glycoconjugate vaccine in liquid formulation remains a challenge for MenA polysaccharide, which similarly to other phosphodiester containing polysaccharides suffers from instability to hydrolysis [72]. Typically, this issue is solved by using lyophilized formulations which are reconstituted with saline before use or by storage at 2–8°C. These solutions, however, implies extra costs for production and distribution of the vaccine, particularly in areas where maintaining the cold chain is not trivial, such as emerging and poor countries.

More stable analogues of MenA polysaccharide have been designed by using C-phosphonate where the phosphodiesters is replacing with a more stable C-phosphonate group (Fig. 4b) [73, 74]. Alternatively, carba-analogues in which the ring-oxygen is replaced by a methylene group, have also been made [75]. Initially, short oligomers of these carbaMenA analogues, containing up to three repeating units, were generated and conjugated to CRM_197_ (Fig. 4c) [76]. The resulting conjugates showed induction of antibodies capable of recognizing the native MenA polysaccharide, but with very poor immunogenicity. More recently, oligomers up to 8 repeating units in length were generated [77]. Particularly, the octamer showed a unique capacity to recognize a bactericidal murine monoclonal antibody that has been mapped by Saturation Transfer NMR and X-ray crystallography, showing that it binds to an O-acetylated trisaccharide epitope [78]. While initial studies with a conjugate of the non-O-acetylated octamer provided unsatisfactory results, introduction of O-acetylation at a level up to 75 %, similar to the natural polysaccharide, generated an antigen which upon conjugation induced bactericidal antibodies similarly to the MenA polysaccharide vaccine benchmark [77]. This work represented a proof-of-principle that glycomimetic vaccines can be used as more stable alternatives to polysaccharide-derived ones.

## Conclusions

Meningococcal glycoconjugate vaccines have been proven very effective medicines to prevent meningitis and drastically reduce incidence of the disease. Successful vaccination with MenC vaccine in UK opened the path to more complex formulations to increase vaccine coverage, which is country variable.

Today we have tetravalent ACWY vaccines in the market and combinations with protein based MenB vaccines under Phase 3 clinical trials. An affordable vaccine against serogroup A has been developed and successfully applied to almost eradicate the disease in the African meningitis belt, thanks to the effort of PATH. A specific ACWXY combination is being developed to cover the X serogroup which caused recent outbreaks in Africa.

This indicates that attention should be paid to monitor emerging serogroups and ensure vaccine effectiveness in the future. Novel approaches have been directed to the identification of new carrier which could improve current formulations and increase the onset of the antibody response or extend the antibody persistence. Also, new methodologies are under investigation to render the cell free vaccine production feasible. Efforts should be directed to methodologies which can ensure scale up also in emerging countries.
